# A case of a metastatic *SDHA* mutated paraganglioma re-presenting twenty-three years after initial surgery

**DOI:** 10.1530/ERC-17-0206

**Published:** 2017-06-26

**Authors:** Ruth T Casey, Benjamin G Challis, Alison Marker, Deborah Pitfield, Heok K Cheow, Ashley Shaw, Soo-Mi Park, Helen L Simpson, Eamonn R Maher

**Affiliations:** 1 Department of Medical GeneticsUniversity of Cambridge and NIHR Cambridge Biomedical Research Centre and Cancer Research UK Cambridge Centre, Cambridge, UK; 2 Department of EndocrinologyCambridge University NHS Foundation Trust, Cambridge, UK; 3 Department of HistopathologyCambridge University NHS Foundation Trust, Cambridge, UK; 4 Department of RadiologyCambridge University Hospitals NHS Foundation Trust, Cambridge, UK; 5 Department of Diabetes and EndocrinologyUniversity College London Hospitals, NHS Foundation Trust, London, UK

## Dear Editor,

**Figure 1:**
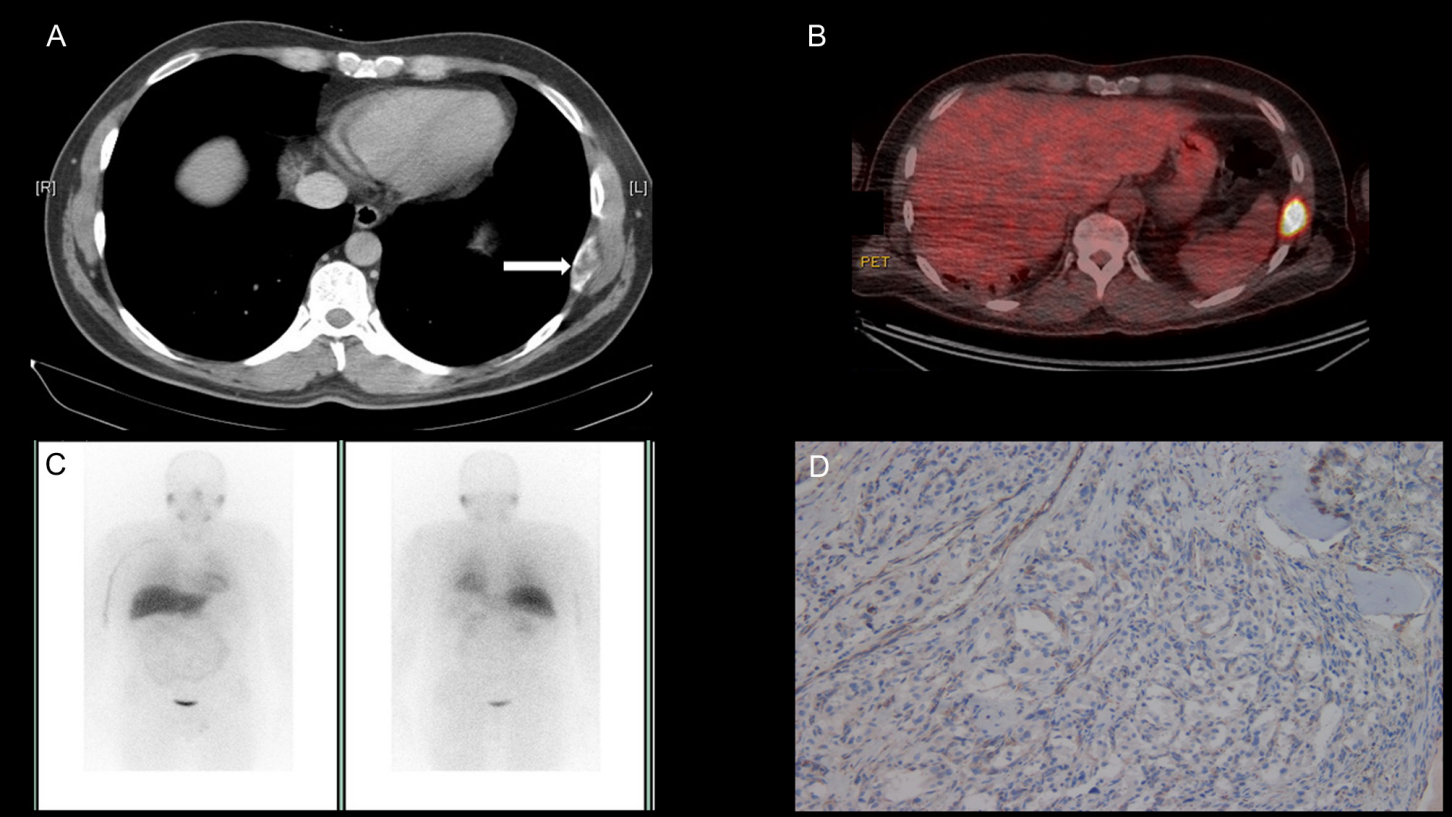
(A) Demonstrates the metastatic deposit in the left 8th rib on CT as indicated by the arrow. (B) Shows the avid rib metastasis on 18F FDG PET CT and (C) shows no tumour avidity on I131 MIBG imaging. (D) Demonstrates loss of SDHB protein expression in the metastatic tumour deposit on SDHB immunostaining indicating SDH deficiency due to the detected germline mutation in *SDHA* (c.91C > T, p.Arg31).

We have read with great interest the recent article by Tufton and coworkers reporting on the risk of metastasis in patients with paraganglioma (PGL) tumours associated with germline SDHA mutations (Tufton *et al*. 2017). Herein, we report a further case of a malignant PGL in a 46-year-old man with a succinate dehydrogenase complex flavoprotein subunit A (*SDHA*) germline mutation (c.91C > T, p.Arg31*). In the case we describe, following the initial surgical removal of a left-sided retroperitoneal PGL, twenty-three years elapsed before the development of a bony metastasis in the eighth left rib. This observation is similar to that of Tufton and coworkers who reported two patients, who developed metastatic disease in 16 and 37 years, respectively, following initial diagnosis (Tufton *et al*. 2017). During investigations for this case, we found that the rib metastasis was avid on 18-fluorodeoxyglucose (FDG) positron emission tomography (PET) computed tomography (CT), but not avid on radiolabeled I131 metaiodobenzylguanidine (MIBG) imaging (Fig. 1). This observation of *SDHA*-related malignant PGL is in keeping with *SDHB*- and *SDHD*-associated PGL, in which reduced avidity on MIBG imaging is due to reduced tumoural expression of noradrenaline transporters (Timmers *et al*. 2007). This clinical report supports the recent literature that suggests a risk of malignancy in patients with SDHA mutated PGL (Bausch *et al*. 2017, Casey *et al*. 2017, Tufton *et al*. 2017). The prolonged time interval between diagnosis and development of metastases in our case suggests that *SDHA*-related tumours are slow growing but it does support long-term surveillance programmes for patients with germline *SDHA* mutations.

Although mutations in *SDHB*, *SDHC* and *SDHD* gene subunits were first identified as predisposition genes for the development of phaeochromocytoma (PC) and PGL tumours over 15 years ago, *SDHA* was first associated with PGL only seven years ago (Burnichon *et al*. 2010) and, more recently, with wild-type gastrointestinal stromal tumours (WT GIST) (Evenepoel *et al*. 2015). It is now recognised that the *SDHA* gene is the most common *SDHx* germline mutation implicated in development of SDH-deficient WT GIST (Boikos *et al*. 2016). Recently, the European-American-Asian Pheochromocytoma-Paraganglioma Registry Study Group reported on 34 index cases with germline *SDHA* mutations and PC or PGL tumours. This group described a high prevalence of head and neck PGL in the *SDHA* cohort 15/34 (44%) and metastatic disease was reported in 4/34 (12%) (Bausch *et al*. 2017).

Here, we report the case of a 46-year-old man who first presented at age 23 years with headache, heat intolerance and abdominal pain. He was subsequently diagnosed with a left-sided retroperitoneal paragangliomama, which was surgically resected. The man had no family history of endocrine tumours and no additional relevant medical history. Following surgery, the patient was surveyed in primary care with annual urinary metanephrine testing. Twenty-three years later he was referred to our specialist neuroendocrine tumour service at Cambridge University Hospital NHS Foundation Trust, due to an elevated urinary normetanpehrine level (urinary normetanephrine 5870 nmol/24 h, reference range 0–4900; urinary metanephrine 756 nmol/24 h, reference range 0–2000), which was first observed 3 months earlier. On review, the patient denied symptoms suggestive of catecholamine excess and, importantly, denied any of the symptoms that he reported at the time of his initial presentation. Review of systems revealed that his only complaint was left side rib pain. Previous investigations in primary care included a plain chest radiograph which did not reveal any abnormality.

We performed plasma metanephrine testing and found an elevated normetanephrine level (2864 pmol/L, reference range <1000 pmol/L) in the context of normal plasma metanephrine (197 pmol/L, reference range <600 pmol/L) and methoxytyramine measurements (95.6 pmol/L, reference range <180 pmol/L). In light of the elevated normetanephrine level in urine and plasma, CT of the neck, thorax, abdomen and pelvis was performed and a metastatic deposit in the eighth left rib with no evidence of additional disease was identified (Fig. 1A). An ^131^I-MIBG scan was performed both for staging purposes and to evaluate suitability for ^131^I-MIBG therapy. Interestingly, the metastatic deposit was not avid (Fig. 1C). An ^18^F-FDG PET CT confirmed avidity (maximum standard uptake value of 19.1) in the left rib lesion (Fig. 1B) but no additional disease was identified. The patient was referred for a thoracotomy and surgical resection of the 8th rib after appropriate pre-operative optimisation with alpha-blockade. Subsequently, histological analysis confirmed the diagnosis of a metastatic paraganglioma with SDHB immunonegativity. SDHA immunohistochemistry was not performed (Fig. 1D).

Next generation sequencing of *SDHA*, *SDHB*, *SDHC*, *SDHD*, *SDHAF2*, *MAX*, TMEM127, *VHL*, *RET* and *FH* was performed on germline DNA from this patient after obtaining informed consent. The Illumina Trusight-One assay was used for sequencing and a mean coverage depth of >20-fold was achieved for 98% of the regions sequenced. Whole exon deletions, duplications and large rearrangements are not detected using this method and multiple ligation probe analysis was performed for *VHL*, *SDHB*, *SDHC* and *SDHD*. This testing identified a pathogenic truncating mutation in the *SDHA* gene (c.91C > T, p.Arg31*), which was confirmed by Sanger sequencing.

Post-operatively, repeat plasma metanephrine levels returned to normal (normetanephrine, 391 pmol/L; metanephrine, 180 pmol/L). This patient will remain under regular clinical follow-up for the development of synchronous or metachronous tumours. Given his germline *SDHA* mutation status and previous disease avidity on ^18^F-FDG PET CT, going forward, this will be the preferred surveillance imaging modality if there is no detectable disease on conventional cross-sectional imaging using CT/MRI, in the presence of elevated plasma metanpehrine or methoxytyramine levels.

In summary, the case we describe provides additional evidence for the risk of metastatic disease in *SDHA* mutated PGL. Moreover, our report highlights the utility of ^18^F-FDG PET CT in the detection of metastatic disease in patients with *SDHA* mutations, as has previously been demonstrated in cases of *SDHB* and *SDHD* related tumours (Timmers *et al*. 2007). Interestingly, and with relevance to optimal surveillance strategies for patients with germline *SDHA* mutations, the significant lag period (23 years) between initial presentation and the development of metastatic disease described in our case was also observed in two other cases of *SDHA* mutated malignant PGL (Tufton *et al*. 2017). The incidence of metastatic disease in *SDHB* related PC and PGL has been reported in two studies as 19% (Benn *et al*. 2006) and 16% (Srirangalingam *et al*. 2008) over a mean follow-up of 48 and 70 months, respectively. These data suggest that the development of metastatic disease may occur earlier in the disease course of *SDHB*-associated PGL/PC. Further study is required to define the risk of malignant disease in *SDHA*-related PGL/PC and the median interval for the development of malignancy. Recent literature suggests that life-long surveillance, as recently recommended by the European Society of Endocrinology (Plouin *et al*. 2016), is crucial for patients with *SDHA* gene mutations, in addition to those patients with mutations in other *SDH* subunits (*SDHB/SDHC/SDHD*). However, we acknowledge that prospective studies with extended follow-up periods of 15–20 years may be necessary to reveal the true incidence of metastasis in *SDHx* related PGL and to stratify individual surveillance protocols for patients based on the SDHx germline subunit mutation.

## Declaration of interest

The authors declare that there is no conflict of interest that could be perceived as prejudicing the impartiality of this article.

## Funding

The authors thank the following funding agencies: NIHR (E R M), European Research Council Advanced Researcher Award (E R M), the British Heart Foundation (E R M) and Health Research Board Ireland (R C).

## References

[bib1] Bausch B Schiavi F Ni Y Welander J Patocs A Ngeow J Wellner U Malinoc A Taschin E Barbon G 2017 Clinical characterization of the pheochromocytoma and paraganglioma susceptibility genes SDHA, TMEM127, MAX, and SDHAF2 for gene-informed prevention. JAMA Oncology [in press]. (10.1001/jamaoncol.2017.0223) PMC5824290 28384794

[bib2] Benn DE Gimenez-Roqueplo AP Reilly JR Bertherat J Burgess J Byth K Croxson M Dahia PL Elston M Gimm O, 2006 Clinical presentation and penetrance of pheochromocytoma/paraganglioma syndromes. Journal of Clinical Endocrinology and Metabolism 91 827–836. (10.1210/jc.2005-1862) 16317055

[bib3] Boikos SA Pappo AS Killian JK LaQuaglia MP Weldon FC Georger S Trent JC von Mehren M Wright JA Schiffman JD 2016 Molecular subtypes of KIT/PDGFRA Wild-type gastrointestinal stromal tumors: a report from the National Institutes Of Health Gastrointestinal Stromal Tumor Clinic. JAMA Oncology 2 922–928. (10.1001/jamaoncol.2016.0256) 27011036 PMC5472100

[bib4] Burnichon N Brière JJ Libé R Vescovo L Rivière J Tissier F Jouanno E Jeunemaitre X Bénit P Tzagoloff A 2010 SDHA is a tumor suppressor gene causing paraganglioma. Human Molecular Genetics 19 3011–3020. (10.1093/hmg/ddq206) 20484225 PMC2901140

[bib5] Casey RT Ascher DB Rattenberry E Izatt L Andrews KA Simpson HL Challis B Park S-M Bulusu VR Lalloo F 2017 SDHA related tumorigenesis: a new case series and literature review for variant interpretation and pathogenicity. Molecular Genetics and Genomic Medicine 5 237–250. (10.1002/mgg3.279) 28546994 PMC5441402

[bib6] Evenepoel L Papathomas TG Krol N Korpershoek E de Krijger R Persu A Dinjens WN 2015 Toward an improved definition of the genetic and tumor spectrum associated with SDH germ-line mutations. Genetics in Medicine 17 610–620. (10.1038/gim.2014.162) 25394176

[bib7] Plouin PF Amar L Dekkers OM Fassnacht M Gimenez-Roqueplo AP Lenders JW Lussey-Lepoutre C Steichen O & Guideline Working Group 2016 European Society of Endocrinology Clinical Practice Guideline for long-term follow-up of patients operated on for a phaeochromocytoma or a paraganglioma. European Journal of Endocrinology 174 G1–G10. (10.1530/EJE-16-0033) 27048283

[bib8] Srirangalingam U Walker L Khoo B MacDonald F Gardner D Wilkin TJ Skelly RH George E Spooner D Monson JP 2008 Clinical manifestations of familial paraganglioma and phaeochromocytomas in succinate dehydrogenase B (SDH-B) gene mutation carriers. Clinical Endocrinology 69 587–596. (10.1111/j.1365-2265.2008.03274.x) 18419787

[bib9] Timmers HJ Kozupa A Chen CC Carrasquillo JA Ling A Eisenhofer G Adams KT Solis D Lenders JW Pacak K 2007 Superiority of fluorodeoxyglucose positron emission tomography to other functional imaging techniques in the evaluation of metastatic SDHB-associated pheochromocytoma and paraganglioma. Journal of Clinical Oncology 25 2262–2269. (10.1200/JCO.2006.09.6297) 17538171

[bib10] Tufton N Ghelani R Srirangalingam U Kumar VKA Drake W Iacovazzo D Skordilis K Berney DM Al-Mrayat M Khoo B 2017 SDHA mutated paragangliomas may be at high risk of metastasis. Endocrine-Related Cancer 24 L43–L49. (10.1530/ERC-17-0030) 28500238

